# Profiles of subgingival microbiomes and gingival crevicular metabolic signatures in patients with amnestic mild cognitive impairment and Alzheimer’s disease

**DOI:** 10.1186/s13195-024-01402-1

**Published:** 2024-02-19

**Authors:** Che Qiu, Wei Zhou, Hui Shen, Jintao Wang, Ran Tang, Tao Wang, Xinyi Xie, Bo Hong, Rujing Ren, Gang Wang, Zhongchen Song

**Affiliations:** 1 Department of Periodontology, Shanghai Ninth People’s Hospital, Shanghai Jiao Tong University School of Medicine; College of Stomatology, Shanghai Jiao Tong University, Zhizaoju Road No.639, Huangpu District, Shanghai, 200011 People’s Republic of China; 2 National Center for Stomatology; National Clinical Research Center for Oral Diseases; Shanghai Key Laboratory of Stomatology; Shanghai Research Institute of Stomatology, Zhizaoju Road No.639, Huangpu District, Shanghai, 200011 People’s Republic of China; 3 Laboratory of Oral Microbiota and Systemic Diseases, Shanghai Ninth People’s Hospital, Shanghai Jiao Tong University School of Medicine; College of Stomatology, Shanghai Jiao Tong University, Jinzun Road No.115, Pudong District, Shanghai, 200125 People’s Republic of China; 4 Department of Neurology and Institute of Neurology, Ruijin Hospital Affiliated to Shanghai Jiao Tong University School of Medicine, Ruijin 2nd Road No.197, Huangpu District, Shanghai, 200025 People’s Republic of China; 5 Department of Geriatric Psychiatry, Shanghai Mental Health Center, Shanghai Jiao Tong University School of Medicine and Alzheimer’s Disease and Related Disorders Center, Shanghai Jiao Tong University, South Wanping Road No.600, Xuhui District, Shanghai, 200030 People’s Republic of China

**Keywords:** Microbiome, Metabolome, Multiomics, Alzheimer’s disease, Mild cognitive impairment, Periodontitis

## Abstract

**Background:**

The relationship between periodontitis and Alzheimer’s disease (AD) has attracted more attention recently, whereas profiles of subgingival microbiomes and gingival crevicular fluid (GCF) metabolic signatures in AD patients have rarely been characterized; thus, little evidence exists to support the oral-brain axis hypothesis. Therefore, our study aimed to characterize both the microbial community of subgingival plaque and the metabolomic profiles of GCF in patients with AD and amnestic mild cognitive impairment (aMCI) for the first time.

**Methods:**

This was a cross-sectional study. Clinical examinations were performed on all participants. The microbial community of subgingival plaque and the metabolomic profiles of GCF were characterized using the 16S ribosomal RNA (rRNA) gene high-throughput sequencing and liquid chromatography linked to tandem mass spectrometry (LC–MS/MS) analysis, respectively.

**Results:**

Thirty-two patients with AD, 32 patients with aMCI, and 32 cognitively normal people were enrolled. The severity of periodontitis was significantly increased in AD patients compared with aMCI patients and cognitively normal people. The 16S rRNA gene sequencing results showed that the relative abundances of 16 species in subgingival plaque were significantly correlated with cognitive function, and LC–MS/MS analysis identified a total of 165 differentially abundant metabolites in GCF. Moreover, multiomics Data Integration Analysis for Biomarker discovery using Latent cOmponents (DIABLO) analysis revealed that 19 differentially abundant metabolites were significantly correlated with *Veillonella parvula*, *Dialister pneumosintes*, *Leptotrichia buccalis*, *Pseudoleptotrichia goodfellowii*, and *Actinomyces massiliensis*, in which galactinol, sn-glycerol 3-phosphoethanolamine, D-mannitol, 1 h-indole-1-pentanoic acid, 3-(1-naphthalenylcarbonyl)- and L-iditol yielded satisfactory accuracy for the predictive diagnosis of AD progression.

**Conclusions:**

This is the first combined subgingival microbiome and GCF metabolome study in patients with AD and aMCI, which revealed that periodontal microbial dysbiosis and metabolic disorders may be involved in the etiology and progression of AD, and the differential abundance of the microbiota and metabolites may be useful as potential markers for AD in the future.

**Supplementary Information:**

The online version contains supplementary material available at 10.1186/s13195-024-01402-1.

## Introduction

Alzheimer’s disease (AD), a degenerative disease of the central nervous system, is characterized pathologically by extracellular β-amyloid containing plaques and intracellular tau-containing neurofibrillary tangles and is the most common cause of dementia [1, 2]. Thus far, the exact molecular mechanism of AD pathogenesis is not fully understood and several hypotheses have been proposed to explain the AD pathological mechanisms [3], of which the infection hypothesis has gained traction and recognition due to new evidence regarding the association of various pathogenic microbes and AD [4]. However, research on the infection hypothesis has mainly focused on the gut microbiota and the gut-brain axis [5, 6], while few studies have focused on the oral microbiota. Recently, a new concept referred to as the oral-brain axis has emerged [7, 8]; nevertheless, the profiles of oral-specific bacterial species/metabolites and their role in the pathogenesis and diagnosis of AD have not yet been established.

It is well known that the oral cavity is anatomically closer to the cerebrum than the gut, and the oral microbiota is the second largest microbiota in the human body [9]. Importantly, as one of the most common chronic inflammatory and infectious oral diseases [10], periodontitis plays a potential role in the development of AD through chronic subgingival microbial dysbiosis [11–13]. Our and others’ previous studies have indicated that the periodontal microbiota may affect the pathological process of AD by activating microglia to induce inflammation or the release of metabolites [11, 14, 15], but the attached and unattached subgingival microbiota in AD patients has not been fully investigated. In addition, as a chronic degenerative disease, the pathological changes of AD may occur in the clinical stage of amnestic mild cognitive impairment (aMCI) before the dementia symptoms [16]. However, changes in the subgingival microbiota of patients with aMCI have not been characterized.

Moreover, bacterial biochemical alterations due to microbial dysbiosis are known to regulate host metabolic pathways. Metabolites are also important functional outputs that affect AD progression after host-microbe interactions in the infection hypothesis [17]. In periodontal tissue, as a type of tissue fluid that flows into gingival crevices, gingival crevicular fluid (GCF) contains many metabolites derived from the host and subgingival microbiota in gingival crevices. Therefore, the metabolites in GCF can reflect the interactions between the host-subgingival microbiota and biochemical metabolism as intermediates [18]. Furthermore, GCF is a noninvasive peripheral body fluid that is anatomically closer to cerebrospinal fluid. Therefore, diagnostic and prognostic biomarker analyses using GCF have the potential to detect pathological changes in patients with AD. Guo et al. [11] compared the subgingival microbial communities of GCF in AD patients and cognitively normal people, but the metabolic profiles of GCF associated with AD progression have not been reported.

Therefore, this study aimed to characterize both the microbial community of subgingival plaque and the metabolomic profiles of GCF in patients with AD and aMCI for the first time, further exploring the oral-brain axis and novel host-microbe interactions linking periodontitis to AD etiology and progression.

## Methods

### Study population

This was a cross-sectional study. Participants with AD or aMCI were recruited from the Department of Neurology, Ruijin Hospital, and the Department of Geriatric Psychiatry, Shanghai Mental Health Center from November 2019 to November 2021. Moreover, cognitively normal people (CN group) were enrolled from the Department of Neurology, Ruijin Hospital, and the Department of Periodontology, Shanghai Ninth People’s Hospital from November 2019 to December 2021. All volunteers provided their informed, written consent before study participation. This study was approved by the Research Ethics Committee of Shanghai Ninth People’s Hospital, and related hospitals (Ethical consent No. SH9H-2019-T218-1).

### Inclusion and exclusion criteria

#### Subjects with the following conditions were included in this study:

- Patients with AD dementia who were diagnosed following the National Institute on Aging and Alzheimer’s Association guidelines for probable AD dementia with the support of magnetic resonance imaging and all patients with aMCI who met the previously published National Institute on Aging and Alzheimer’s Association criteria (2011) for “MCI due to AD,” with memory deficits as the only complaint of cognitive impairment, reported by the patient, caregiver, or physician [19];
- Cognitively normal people with a Montreal Cognitive Assessment (MoCA) score of ≥ 26 and a Mini-Mental State Examination (MMSE) score of ≥ 27 [20];
- Those with a functional tooth number of ≥ 6;
- Participants who were able to cooperate during periodontal examination;
- Patients or their legal guardians signed the informed consent forms.

#### Subjects who met any of the following criteria were excluded from this study

- Diagnosed with other types of dementia;
- Hamilton Depression Scale score of > 6 [21];
- Functional tooth number of < 6;
- Received periodontal treatments for 6 months before sampling;
- Had open surgical treatments of the head and/or mouth;
- Diagnosed with acquired immune deficiency syndrome, hepatitis B infection, or other infectious diseases;
- Diagnosed with diabetes mellitus;
- Those with a history of cancer, staphylococcal toxic shock syndrome, inherited metabolic diseases, autoimmune diseases, hormone-dependent diseases, radiotherapy, and chemotherapy;
- Those administered antibiotics, immunomodulators, cytokines, and probiotics within the last 3 months.

### Clinical examination

#### Neurological examination

All participants underwent a set of standardized neurological examinations by two or three neurologists who specialized in dementia and were eligible to the above-reported diagnostic criteria [19, 22]. Data were also gathered on age, sex, educational level, and family and personal histories related to cognitive impairment. After the neurological examination, patients were classified as having aMCI or AD. For diagnostic and prognostic evaluation, some participants also underwent 8F-florbetapir (AV45) positron emission tomography (PET) and apolipoprotein E (*APOE*) gene testing after obtaining informed consent.

#### Periodontal examination

A full-mouth comprehensive examination of the periodontal condition was performed for all individuals by two periodontists from the Department of Periodontology, Shanghai Ninth People’s Hospital, including the number of teeth, periodontal probing depth (PPD), clinical attachment level (CAL), percentage of CAL > 3 mm, gingival index (GI), plaque index (PLI), and percentage of bleeding on probing (BOP). Specifically, the number of teeth, CAL, and GI index reflected the severity of periodontitis, PPD reflected the complexity of periodontitis, while CAL > 3 mm% and BOP% can reflect the extent and distribution of periodontal inflammation, and PLI reflects the oral hygiene status of the subjects [13]. Data consistency of periodontal examination was assessed by the Kappa coefficient and inter-class correlation coefficients [23].

### Sample collection

After clinical examination, subgingival plaque and GCF samples were obtained from Ramfjord index teeth [24, 25] (the maxillary right and mandibular left first molars, maxillary left and mandibular right first premolars, and maxillary left and mandibular right central incisors) and teeth with moderate and deep periodontal pockets (PPD > 3 mm). Before sample collection, teeth were isolated using sterile cotton rolls and air-dried. The supra-gingival plaque was removed, and the subgingival plaque (including the attached and unattached subgingival microbiota) was scraped with Gracey curettes and placed in a sterile Eppendorf tube containing 0.5 ml of 1 × phosphatic buffer solution (pH 7.2) and stored at − 80 °C. Three or four sterile dental absorbent paper points on each site per tooth were gently inserted into the gingival sulcus or periodontal pockets for approximately 30 s to obtain GCF, which was then weighed using a Mettler analytical balance to a sensitivity of 0.1 mg (Mettler Toledo, Shanghai, China), placed in sterile Eppendorf tubes on ice, transported to the laboratory and stored at − 80 °C. A total of 20–40 strips were collected from each participant. Both subgingival plaque and GCF samples were collected prior to clinical measurements, and samples visibly contaminated with blood were discarded.

### Microbial DNA extraction and 16S ribosomal RNA (rRNA) sequencing

#### DNA extraction and 16S rRNA gene amplicon sequencing

Total genomic DNA was extracted from subgingival plaque samples using the OMEGA DNA Kit (M5635-02) (Omega Bio-Tek, Norcross, GA, USA) following the manufacturer’s instructions and stored at − 20 °C prior to further analysis. The quantity and quality of the extracted DNA were measured using a NanoDrop NC2000 spectrophotometer (Thermo Fisher Scientific, Waltham, MA, USA) and agarose gel electrophoresis, respectively. Polymerase chain reaction (PCR) amplification of the nearly full-length bacterial 16S rRNA genes was performed using the forward primer 27F (5′-AGAGTTTGATCCTGGCTCAG-3′) and the reverse primer 1492R (5′-ACCTTGTTACGACTT-3′). The extracted DNA was amplified with two-step PCR, with sample-specific 16-bp barcodes incorporated into the forward and reverse primers for multiplex sequencing in the second PCR step. Thermal cycling consisted of initial denaturation at 98 °C for 2 min, followed by 25/10 cycles (for the first and second amplification steps, respectively) consisting of denaturation at 98 °C for 30 s, annealing at 55 °C for 30 s, and extension at 72 °C for 90 s, with a final extension of 5 min at 72 °C. All PCR amplicons were purified with Agencourt AMPure Beads (Beckman Coulter, Indianapolis, IN) and quantified using the PicoGreen dsDNA Assay Kit (Invitrogen, Carlsbad, CA, USA). After the individual quantification step, amplicons were pooled in equal amounts, and single-molecule real time sequencing technology was performed using the PacBio Sequel platform at Shanghai Personal Biotechnology Co., Ltd (Shanghai, China).

#### Bioinformatics and data analysis

Microbiome bioinformatics was mainly performed using the QIIME2 and R packages (v3.2.0). α-Diversity indices, such as the Chao1 richness estimator, observed species, Shannon diversity index, Simpson index, Faith’s PD, Pielou’s evenness and Goods coverage, were calculated using the amplicon sequence variant (ASV) table in QIIME2 and visualized as box plots. ASV-level ranked abundance curves were generated to compare the richness and evenness of ASVs among samples. β-Diversity analysis was performed to investigate the structural variation in microbial communities across samples using Bray–Curtis metrics and visualized via principal coordinate analysis (PCoA). The significance of the differentiation of microbiota structure among groups was assessed by permutational multivariate analysis of variance and analysis of similarities using QIIME2. The differential species compositions and abundances were visualized using heatmaps, and linear discriminant analysis effect size (LEfSe) was performed to detect differentially abundant taxa across groups using the default parameters. The functional microbes that primarily drive the difference in the severity of cognitive impairment (based on the groups, MoCA and MMSE scores) among the CN, aMCI, and AD groups were determined using microbiome multivariable associations with linear models (MaAsLin2).

#### Data access

All microbial raw sequences were deposited in the NCBI Sequence Read Archive under accession number PRJNA903172.

### GCF sample preparation and metabolic analysis

#### GCF sample preparation and liquid chromatography linked to tandem mass spectrometry (LC–MS/MS) data acquisition

To extract metabolites from GCF samples, 40 μl of cold extraction solvent methanol/acetonitrile/H_2_O (2:2:1, v/v/v) was added to each 10-mg sample (each sample had eight to ten dental absorbent paper points, and the weight of GCF was calculated) and adequately vortexed. After vortexing, the samples were incubated on ice for 20 min and then centrifuged at 14,000 × *g* for 20 min at 4 °C. The supernatant was collected and dried in a vacuum centrifuge at 4 °C. For LC–MS/MS analysis, the samples were redissolved in 100 μl acetonitrile/water (1:1, v/v) solvent and transferred to LC vials. Extracts were analyzed using a quadrupole time-of-flight mass spectrometer (Sciex TripleTOF 6600) coupled to hydrophilic interaction chromatography via electrospray ionization for untargeted metabolomics of polar metabolites at Shanghai Applied Protein Technology Co., Ltd. (Shanghai, China). The mass spectrometer was operated in both negative ion and positive ionization modes. The product ion scan was acquired using information-dependent acquisition with the high sensitivity mode selected.

#### Bioinformatics and data analysis

The metabolites were identified by accuracy mass (< 25 ppm) and secondary mass spectrometry data, which were matched with the standard database (Shanghai Applied Protein Technology Co., Ltd.). In the extracted ion features, only the variables having more than 50% of the nonzero measurement values in at least one group were kept. Compound identification of metabolites by MS/MS spectra was performed with an in-house database established with available authentic standards. After normalization to total peak intensity, the processed data were uploaded before importing into SIMCA-P (version 14.1, Umetrics, Umea, Sweden), where they were subjected to multivariate data analysis, including pareto-scaled principal component analysis (PCA) and partial least-squares discriminant analysis (PLS-DA). Significance was determined using MaAsLin2, and a heatmap was presented as a visual aid for the clustering of differentially abundant metabolites and pathways. The Euclidean distance algorithm for similarity measurement and the average linkage clustering algorithm (clustering uses the centroids of the observations) for clustering were selected when performing hierarchical clustering. A heatmap is presented as a visual aid for clustering differentially abundant metabolites and pathways. For the Kyoto Encyclopedia of Genes and Genomes (KEGG) pathway annotation, the metabolites were blasted against the online KEGG database (KEGG; http://www.genome.jp/kegg/). The corresponding KEGG pathways were then extracted. To further explore the impact of differentially expressed metabolites, enrichment analysis was performed. KEGG pathway enrichment analyses were performed using the differentially abundant metabolites of each pathway as the background dataset.

### Correlations of the microbiome, metabolome, and clinical indices

#### Integrative analysis of the microbiome and metabolome

A multiomics integrative method named Data Integration Analysis for Biomarker discovery using Latent cOmponents (DIABLO) was used to discriminate between the CN, aMCI, and AD groups [26] and to further identify the optimal biomarkers from functional microbes and differentially abundant metabolites. DIABLO is implemented in the mix-Omics R Bioconductor package with functions for parameter choice and visualization to assist in the interpretation of integrative analyses.

#### Association between candidate biomarkers and clinical indices

A heatmap of Spearman’s rank correlation coefficient was used to illustrate the relationships between candidate biomarkers from the DIABLO analysis and clinical indices.

#### Diagnostic capability of potential biomarkers

Values for the area under the curve (AUC) of the receiver operating characteristic (ROC) curve were used to assess the ability of candidate biomarkers to diagnosis and indicate clinical progression of AD.

### Statistical analysis

Continuous variables were expressed as the mean ± standard deviation (SD). Analysis of demographics and clinical characteristics among the CN, aMCI, and AD groups was conducted via analysis of variance (ANOVA) test or Kruskal–Wallis test for continuous variables, and chi-square tests for categorical variables using SPSS version 26.0 (SPSS, Inc., Chicago, IL, USA) and GraphPad Prism 6 (GraphPad Software, Inc.). Continuous variables were also adjusted for age and sex using linear regression analysis. Statistical significance was set at *P* < 0.05. The functional microbes and metabolites that primarily drive the difference among the CN, aMCI, and AD groups were determined using MaAsLin2, and statistical significance was set at an FDR-corrected *P* value of < 0.25. The correlations between the microbiome, metabolome, and clinical indices were analyzed using DIABLO or Spearman’s rank correlation coefficient. The statistical significance was set at a correlation coefficient of *r* > 0.3.

## Results

### Demographic and clinical characteristics of the participants

In total, 32 individuals with AD (including 8 mild, 17 moderate, and 7 severe according to the MMSE), 32 individuals with aMCI (aMCI group), and 32 cognitively normal people (CN group) were enrolled in this study. No significant differences were found in years of education, marital status, or living status among the three groups (Table 1). The mean MoCA and MMSE scores in the AD group were 9.56 ± 6.44 and 12.56 ± 6.44, respectively, which were much lower than those of the aMCI and CN groups, and the MoCA and MMSE scores in the aMCI group were also significantly lower than those in the CN group after adjusting for age and sex (Table 1). Moreover, periodontal examination showed that the number of teeth in the CN group was significantly greater than that in the AD group, while the CAL, CAL > 3 mm%, PLI, and BOP% in the AD group were significantly higher than those in the CN group, and the CAL and CAL > 3 mm% in the AD group were significantly greater than those in the aMCI group after adjusting for age and sex. However, there was no significant difference in the PPD and GI among the AD, aMCI, and CN groups (Table 1).

**Table 1:** Participants’ demographic features and clinical characteristics

	CN (***n*** = 32)	aMCI (***n*** = 32)	AD (***n*** = 32)	***P*-value**	**Age- and sex-adjusted *P*-value**
**Demographics**					
Age (mean ± SD) years	65.75 ± 6.33	72.31 ± 8.07	76.03 ± 8.23	**0.0000**^**a,b**^	**/**
Male (%)	10 (31.25)	9 (28.13)	20 (62.50)	**0.0083**^**b,c**^	**/**
Female (%)	22 (68.75)	23 (71.87)	12 (37.50)	**/**	**/**
Education > 6 years (%)	29 (90.63)	30 (93.75)	33 (96.88)	0.5866	/
**Marital status**					
Unmarried (%)	0 (0)	0 (0)	0 (0)	0.1184	/
Married (%)	32 (100)	30 (93.75)	28 (87.50)	/	/
Divorced (%)	0 (0)	0 (0)	0 (0)	/	/
Widowed (%)	0 (0)	2 (6.25)	4 (12.50)	/	/
**Living status**					
Living alone (%)	3 (9.37)	1 (3.13)	2 (6.25)	0.1120	/
Living with spouse (%)	27 (84.38)	24 (75.00)	20 (62.50)	/	/
Living with children (%)	2 (6.25)	5 (15.62)	4 (12.50)	/	/
Other (%)	0 (0)	2 (6.25)	6 (18.75)	/	/
**Neurological indices**					
MoCA (mean ± SD)	27.25 ± 1.50	18.88 ± 5.35	9.56 ± 6.44	**0.000**^**a,b,c**^	**0.000**^**a,b,c**^
MMSE (mean ± SD)	28.34 ± 1.07	24.13 ± 4.29	12.56 ± 6.44	**0.000**^**a,b,c**^	**0.000**^**a,b,c**^
*APOE* gene test (e4 carrier frequency%)	1 (0)	14/5 (35.71)	8/6 (75.00)	/	/
AV45 PET (positive rate%)	0 (0)	5/3 (60.00)	8/8 (100.00)	/	/
**Periodontal indices**					
Number of teeth	25.88 ± 3.25	21.31 ± 6.78	18.47 ± 7.76	**0.000**^**a,b**^	**0.023**^**b**^
PPD (mean ± SD) mm	2.56 ± 0.68	2.65 ± 0.52	3.05 ± 0.83	**0.016**^**b**^	0.057
CAL (mean ± SD) mm	3.09 ± 0.95	3.64 ± 0.99	4.61 ± 1.42	**0.000**^**b,c**^	**0.009**^**b,c**^
CAL > 3 mm (mean ± SD) %	35.72 ± 24.52	48.36 ± 24.28	69.44 ± 26.31	**0.000**^**b,c**^	**0.007**^**b,c**^
GI (mean ± SD)	1.67 ± 0.24	1.70 ± 0.22	1.81 ± 0.25	0.048	0.302
PLI (mean ± SD)	1.84 ± 0.26	2.03 ± 0.31	2.22 ± 0.44	**0.000**^**b**^	**0.013**^**b**^
BOP (mean ± SD) %	48.91 ± 24.21	65.42 ± 19.78	72.18 ± 29.68	**0.002**^**b**^	**0.034**^**b**^

ANOVA test or Kruskal–Wallis test were used for continuous variables, and chi-square tests was used for categorical variables. Adjustment for age and sex was performed using linear regression analysis. “a” means CN group and aMCI group are significantly different, “b” means CN group and AD group are significantly different, and “c” means aMCI group and AD group are significantly different

### The structure and diversity of the subgingival microbiota of the participants

To compare the composition of the subgingival microbiota between the AD, aMCI, and CN groups, we profiled the subgingival microbiota composition using 16S rRNA amplicon high-throughput sequencing.

**Fig. 1:**
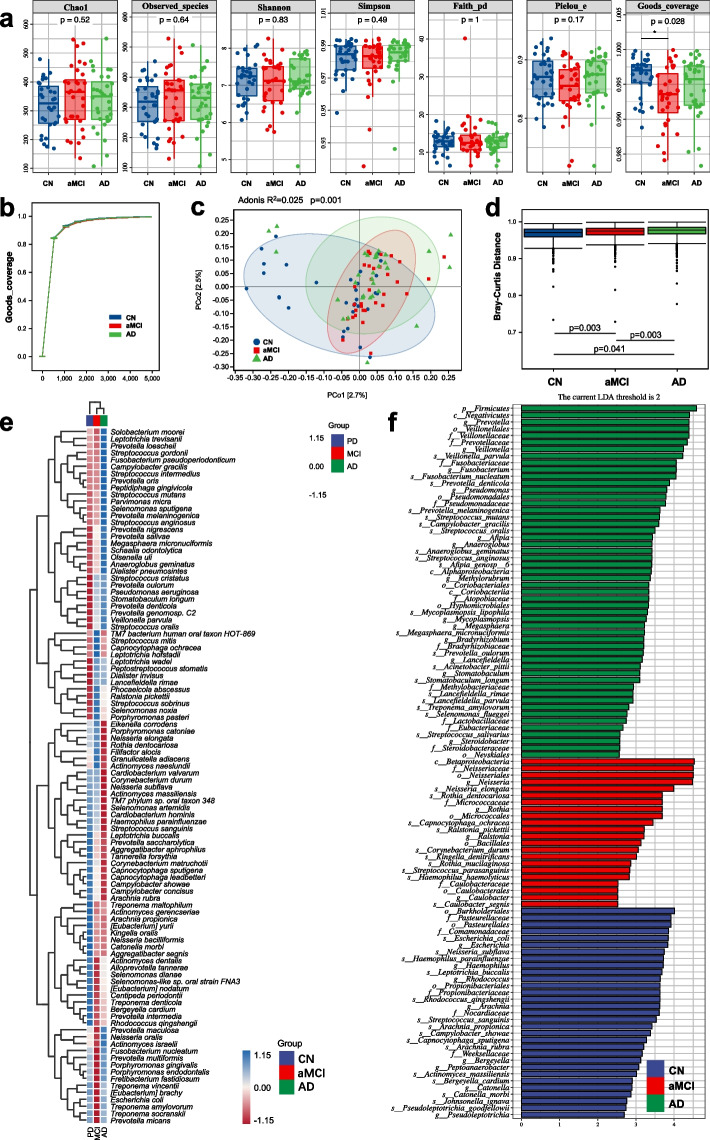
Different structures and diversity of the subgingival microbiota in the CN, aMCI, and AD groups.** a** The α-diversity indices Chao1, observed species, Shannon, Simpson, Faith’s PD, Pielou’s evenness, and Goods coverage. Statistical significance was set at *P* < 0.05. **b** Rarefaction curve of the Goods coverage index. With increasing sequencing depth, the Goods coverage index of the observed samples was close to saturation. **c** The structural shifts (β-diversity) presented by the Bray–Curtis PCoA plot based on the ASV abundance. The *x*-coordinate represents the first principal coordinate, the *y*-coordinate represents the second principal coordinate, and the percentage represents the influence rate of the principal coordinates on the sample differences. **d** Differences among the three groups. The statistical methods of **c** and **d** were both Adonis analysis. Data are presented as the mean ± SD. Statistical significance was set at *P* < 0.05.** e** The heatmap shows the comparisons of the subgingival microbial community in cognition-normal people and patients with AD and aMCI at the species level (top 100 species). The *x*-coordinate represents the names of the groups; the *y*-coordinate represents the taxon at the species level. **f** LEfSe analysis revealed significant bacterial differences in subgingival microbiota among the CN, aMCI, and AD groups. The LDA scores (log10) > 2 and *P* < 0.05 are listed

Regarding the subgingival microbiota composition with 16S rRNA amplicon high-throughput sequencing, α-diversity analysis showed that there was no significant difference found in the Chao1, observed species, Shannon, Simpson, Faith’s PD, and Pielou’s evenness among the three groups except for the Goods coverage index (Fig. 1a), and the rarefaction curve showed that with increasing of sequencing depth, the Goods coverage index of the observed samples from all 3 groups tended to saturate and approach 1 (Fig. 1b). β-Diversity was presented using the Bray–Curtis PCoA plot based on the ASV abundance, which illustrated that distinct clusters were formed among the three groups, and the results of the differences analysis showed that there were significant differences in microbial structural shifts among the three groups (Fig. 1c, d). To further compare the different compositions among the three groups at the species level, we used cluster heatmaps and the LEfSe method. The heatmaps illustrated significant differences in the relative abundance of bacteria at the species level in the subgingival plaque of subjects among the AD, aMCI, and CN groups (Fig. 1e). We further compared the species composition among the AD, aMCI, and CN groups based on the linear discriminant analysis effect size method. Considering differences in the taxa at the species level with a logarithmic linear discriminant analysis score of > 2.0 and a *P* value of < 0.05, we found that the abundance of 19 species was higher in the AD group, that of 15 species was higher in the CN group, and that of 10 species was higher in the aMCI group (Fig. 1f).

Next, MaAsLin2 analysis revealed that 16 species were associated with cognitive function. Among them, *Veillonella parvula* (*V. parvula*), *Lancefieldella parvula* (*L. parvula*), *Prevotella melaninogenica* (*P. melaninogenica*), *Megasphaera micronuciformis* (*M. micronuciformis*), *Anaeroglobus geminatus* (*A. geminatus*), *Streptococcus anginosus* (*S. anginosus*), *Campylobacter gracilis* (*C. gracilis*), and *Dialister pneumosintes* (*D. pneumosintes*) were negatively correlated with cognitive function, and *[Eubacterium] yurii*, *Pseudoleptotrichia goodfellowii* (*P. goodfellowii*), *Campylobacter rectus* (*C. rectus*), *Leptotrichia buccalis* (*L. buccalis*), *Streptococcus sanguinis* (*S. sanguinis*), *Actinomyces massiliensis* (*A. massiliensis*), *Haemophilus parainfluenzae* (*H. parainfluenzae*) and *Campylobacter concisus* (*C. concisus*) were positively correlated with cognitive function (Table S1).

### Metabolic profiles of GCF samples of the participants

#### Multivariate data analysis and clustering of GCF metabolites

**Fig. 2:**
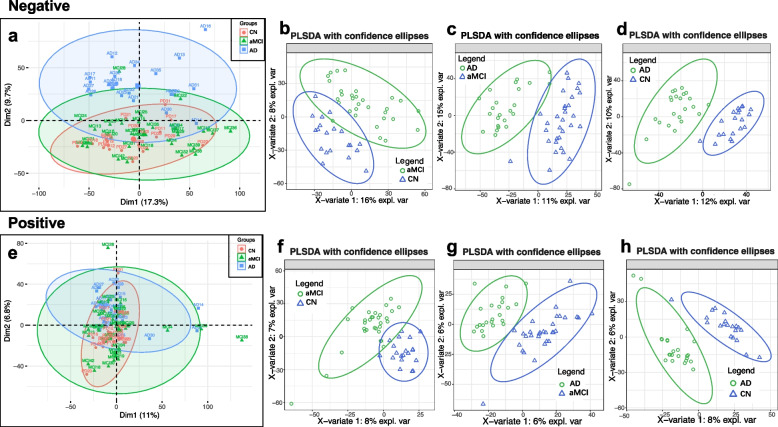
Typical plots of multivariate statistical analysis. PCA and PLS-DA of the metabolic profiles of GCF samples from the AD, aMCI, and CN groups. **a**,** e** PCA plot model of GCF. **b–d**,** f–h** The PLS-DA model of GCF (negative mode: **b–d**; positive mode: **f–h**). The predictions of multivariate statistical analysis show a clear discrimination among the CN, aMCI, and AD groups

To first reveal the profiles of the GCF metabolome, we performed nontargeted metabolomics profiling of 74 GCF samples from the AD, aMCI, and CN groups. After removing the internal standards and pseudopositive peaks and combining the peaks from the same metabolite, 1116 positive-mode features and 686 negative-mode features were identified for use in the subsequent analysis. Multivariate analysis among the AD, aMCI, and CN groups was performed with PCA and PLS-DA. In the plots of PCA scores, we noted a trend of divergence in GCF sample results among the three groups (Fig. 2a, e). The PLS-DA models clearly separated the metabolic profiles of the three groups (Fig. 2b–d, f–h).

In both positive and negative modes, a total of 165 differentially abundant metabolites were identified among the AD, aMCI, and CN groups using MaAsLin2, based on an FDR-corrected *P* value of < 0.25. We further classified them according to their participating pathways or functions through KEGG database annotation, and hierarchical classification was also conducted according to the KEGG metabolic pathways with different metabolites involved, resulting in the identification of 153 metabolic pathways based on the KEGG database. The heat plot of the hierarchical clustering results among the three groups showed that differentially abundant metabolites in the AD group had significantly different patterns of abundance compared to those in the aMCI and CN groups (Figure S1).

#### KEGG enrichment analysis

After annotating the differentially abundant metabolites, enrichment analysis of their KEGG pathways was conducted (Figure S2). Enrichment analysis is usually performed to assess whether a group of metabolites have been present at a certain functional node, which can identify the biological processes most related to biological phenomena.

Several pathways contained more types of differentially abundant metabolites, such as purine metabolism, amino sugar and nucleotide sugar metabolism, lysine degradation, galactose metabolism, phenylalanine, tyrosine and tryptophan biosynthesis, aminoacyl-tRNA biosynthesis, and pyrimidine metabolism (Table S2).

### Correlations of the microbiome, metabolome, and clinical indices

#### Integrative analysis of the microbiome and metabolites

To explore whether the abundance of GCF metabolites correlated with the subgingival microbiota, the covariation among the three groups was investigated using a multiomics method named DIABLO. Notably, most metabolite levels that were elevated in patients with AD, such as 1 h-indole-1-pentanoic acid, 3-(1-naphthalenylcarbonyl)-, sn-glycerol 3-phosphoethanolamine, L-iditol, D-mannitol, galactinol, and adenine, were positively correlated with the majority of AD-enriched species (*D. pneumosintes* and *V. parvula*). In contrast, the enriched species (*P. goodfellowii*, *L. buccalis*, and *A. massiliensis*) in cognitively normal people were negatively correlated with AD-enriched metabolites and positively correlated with N,n-diethyl-2-aminoethanol depletion in these subjects (Figure S3-S5). In summary, these results suggest that the altered subgingival microbiota is related to subgingival metabolism to some extent and that the levels of GCF metabolites may reflect changes in the abundance of these corresponding species. The results of DIABLO (Figure S3-S5) also showed a strong positive contribution of 19 metabolites (Table S3) and five species to the discrimination among the CN, aMCI, and AD groups, suggesting their possible application as indicators of cognitive impairment severity.

#### Relationships between differentiated species/metabolites and clinical phenotypes

**Fig. 3:**
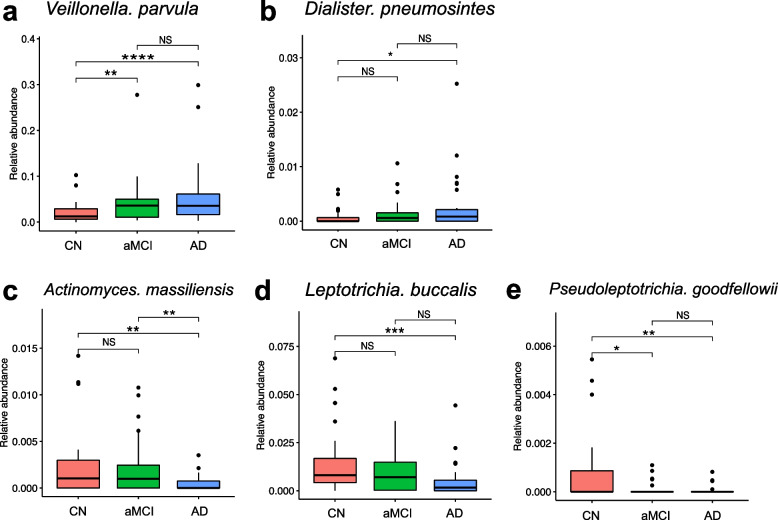
Relative abundances of candidate microbial biomarkers. Taxon abundances at the species level were statistically compared in the CN, aMCI, and AD groups. *P < 0.05, **P < 0.01, ***P < 0.001 and ****P < 0.0001

**Fig. 4:**
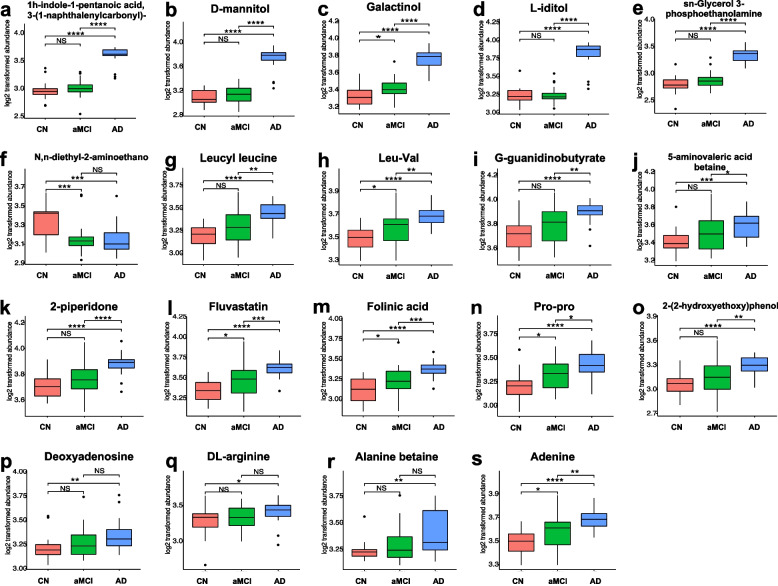
Abundances of candidate metabolic biomarkers of aMCI and AD. *P < 0.05, **P < 0.01, ***P < 0.001, and ****P < 0.0001

The DIABLO results mentioned above showed a strong positive contribution of 19 metabolites (Table S3) and five species to the discrimination among the CN, aMCI, and AD groups, and the relative abundance of these potential biomarkers is shown in Figs. 3 and 4.

**Fig. 5:**
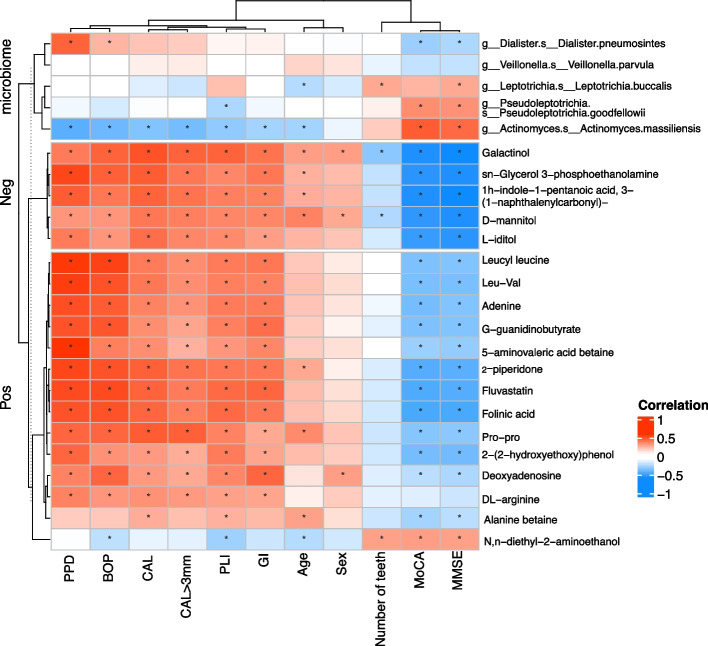
Correlations between candidate biomarkers and clinical indices. Heatmap of candidate biomarkers with clinical indices. Spearman’s rank correlation between five differential species, 19 differentially abundant metabolites, and 11 clinical indices. Blue and red classes denote negative correlation and positive correlation, respectively. *Correlation coefficient *r* > 0.3

Regarding species, as shown in the heatmap of Fig. 5, there was a statistically significant negative correlation between *D. pneumosintes* and the scores of cognitive scales, including MoCA and MMSE, and a statistically significant positive correlation between *D. pneumosintes* and the clinical phenotypes of periodontitis, including PPD and BOP%. In contrast, the enriched species (*P. goodfellowii*, *L. buccalis*, and *A. massiliensis*) in cognitive normal people were negatively correlated with the clinical phenotypes of periodontal condition and positively correlated with the clinical data of cognitive function. Moreover, AD-enriched metabolites such as galactinol, sn-glycerol 3-phosphoethanolamine, 1 h-ndole-1-pentanoic acid, 3-(1-naphthalenylcarbonyl)-, D-mannitol, L-iditol, leucyl leucine, leu-val, adenine, G-guanidinobutyrate, 5-aminovaleric acid betaine, 2-piperidone, fluvastatin, folinic acid, pro-pro, 2-(2-hydroxyethoxy)phenol, deoxyadenosine, DL-arginine, and alanine betaine were negatively correlated with the clinical phenotypes of cognitive function and positively correlated with the periodontal indices, including the PPD, BOP%, CAL, CAL > 3 mm%, PLI, and GI. In contrast, N,n-diethyl-2-aminoethanol was positively correlated with the MoCA and MMSE scores and the number of teeth, but negatively correlated with the PLI and BOP%.

#### Diagnostic value of GCF metabolites for cognitive impairment

**Fig. 6:**
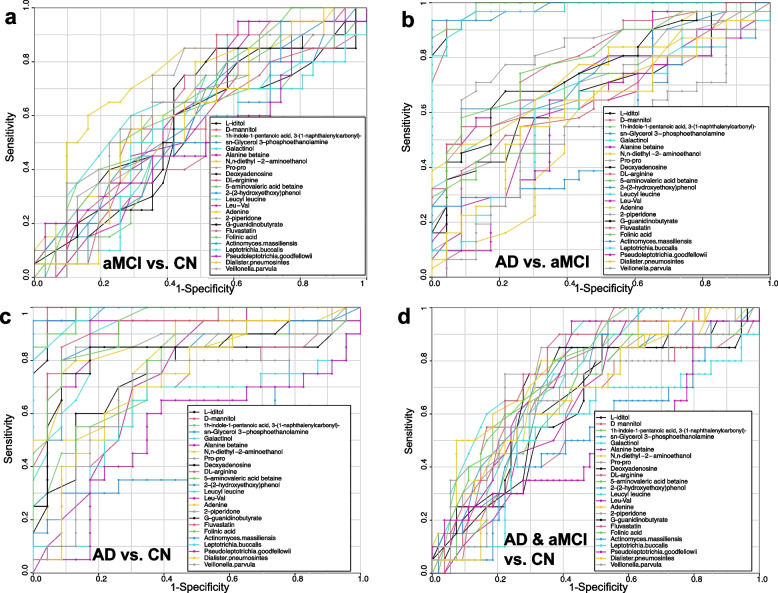
ROC curve of candidate biomarkers for distinguishing the CN group, aMCI group, and AD group. **a–d** ROC curve of 19 differentially abundant metabolites and five differential species for distinguishing the CN group, aMCI group, and AD group

The diagnostic abilities of the DIABLO models were further demonstrated by ROC curve analysis, which showed that galactinol (AUC = 0.98 in AD vs. aMCI/CN), sn-glycerol 3-phosphoethanolamine (AUC = 0.98 in AD vs. aMCI and AUC = 0.99 in AD vs. CN), D-mannitol (AUC = 0.98 in AD vs. aMCI and AUC = 0.99 in AD vs. CN), 1 h-indole-1-pentanoic acid, 3-(1-naphthalenylcarbonyl)- (AUC = 0.99 in AD vs. aMCI and AUC = 0.98 in AD vs. CN), and L-iditol (AUC = 0.99 in AD vs. aMCI/CN) had satisfactory accuracy for the diagnosis of AD (Fig. 6, Table S4), which may indicate that these metabolites may be useful as potential markers of cognitive status. In summary, these results provide insight into novel host-microbe interactions that integrates the metabolic signatures of subgingival dysbiotic communities from AD patients with periodontitis and identifies potential markers of AD progression.

## Discussion

Periodontitis is one of the most common oral chronic inflammatory diseases characterized by the loss of periodontal tissue support, manifested through CAL and radiographically assessed alveolar bone loss, presence of periodontal pocketing, gingival bleeding, and tooth loss [13]. As the initiating factor underlying periodontitis, the composition of dental plaque, especially subgingival microbiota, remains in dynamic balance in a healthy state, while dysbiosis of the subgingival microbiota ecology can lead to inflammation of the periodontal tissues [27, 28]. Previous studies have shown that some subgingival microbes such as *Porphyromonas gingivalis* (*P. gingivalis*) can contribute to microglia activation, Aβ accumulation, and cognitive impairment [14, 15]. Based on the association between periodontitis and AD, oral-brain axis gains traction gradually [3, 7, 8], which suggests that the oral microbiota and their products can affect the brain either directly invading the central nervous system or indirectly through mediating systemic inflammation.

In this study, we combined the periodontal subgingival microbiome and GCF metabolome in patients with AD and aMCI for the first time and successfully screened 16 microbial species that differed significantly in subgingival plaque samples that were significantly correlated with cognitive function and 165 differentially abundant metabolites in GCF samples. Multiomics DIABLO and Spearman’s correlation analysis revealed that the abundance of 19 differentially abundant metabolites in GCF was significantly correlated with five differentially abundant species (*V. parvula*, *D. pneumosintes*, *L. buccalis*, *P. goodfellowii*, and *A. massiliensis*) and neurological/periodontal indices, in which galactinol, sn-glycerol 3-phosphoethanolamine, D-mannitol, 1 h-indole-1-pentanoic acid, 3-(1-naphthalenylcarbonyl)- and L-iditol yielded satisfactory accuracy (AUC > 0.98) for the potential markers of AD and further provided a research base for the oral-brain axis hypothesis.

### Periodontal conditions and cognitive function

According to the results of the Fourth National Oral Health Survey in China, the prevalence of BOP was 88.4% among those aged 55 to 64 years and 82.6% among those aged 65 to 74 years, likely offsetting the poor periodontal health induced by AD [29]. Although previous studies reported conflicting results [30, 31], our results are consistent with those of Martande’s study [30], which found that the severity of periodontitis was correlated with the severity of cognitive impairment in participants. After adjusting for age and sex ratio, CAL and CAL > 3 mm% remained significantly higher in the AD group than in the CN and aMCI groups. Periodontal indicators did not show statistically significant differences in one study with a relatively small sample size [31].

### Microbial community characteristics

In the current study, β-diversity analysis rather than α-diversity analysis of subgingival microbial community comparisons revealed significant differences in the microbial community among samples from cognitively normal individuals, aMCI, and AD patients. One possible explanation is that all individuals in this study were from the same living area (Shanghai, China), which led to nonsignificant differences in bacterial α diversity among the three groups owing to the high similarity of diet and living habits. As cognitive function changes, the composition of the subgingival plaque community shifts in periodontitis patients. At the species level, the microbiota of aMCI and AD patients had higher proportions of *L. parvula*, *P. melaninogenica*, *M. micronuciformis*, *A. geminatus*, *V. parvula*, *S. anginosus*, *C. gracilis*, and *D. pneumosintes*, whereas the proportions of *[Eubacterium] yurii*, *P. goodfellowii*, *C. rectus*, *L. buccalis*, *S. sanguinis*, *A. massiliensis*, *H. parainfluenzae*, and *C. concisus* were higher in the microbiota from cognitively normal individuals. Moreover, the abundance of *P. gingivalis* and *S. sanguinis* was correlated with the severity of periodontitis.

The differential enrichment of these species contributes to the differences in the diversity of subgingival plaque samples and, to a certain extent, to the function of the subgingival microbial community among cognitively normal individuals, aMCI patients, and AD patients. For instance, we found that *V. parvula* from the phylum Firmicutes may be closely related to the progression of both AD and periodontitis. *V. parvula* is an anaerobic gram-negative coccus that is part of the normal flora of the oral cavity and is an important pathogen in periodontitis. An in vivo study showed that the abundance of *V. parvula* was significantly elevated in the oral plaque biofilms of an experimental rat periodontitis model [32]. As an early colonizer, *V. parvula* is also associated with oral malodor [33] and coaggregation with other important periodontal pathogens, such as *Fusobacter nucleatum* (*F. nucleatum*) and *P. gingivalis* [34, 35]. Furthermore, *V. parvula* is an opportunistic pathogen in intracranial infections [36]. *V. parvula* may enter the brain through the blood circulation and stimulate microglia through pathogenic factors, such as lipopolysaccharide, to cause neuroinflammation and eventually lead to AD. Some recent clinical studies also compared the subgingival microbial communities of patients with AD and cognitively normal participants and found a significant increase in the relative abundance of Firmicutes and *V. parvula* in AD patients [11, 37]. Other differential species that have not yet been reported may play a significant role in AD development, such as *D. pneumosintes*. Studies have shown that *D. pneumosintes* and other periodontal pathogens such as *Aggregatibacter actinomycetemcomitans* (*A. actinomycetemcomitans*) have a synergistic relationship in the formation of periodontal plaque biofilms, which could influence PPD and CAL, thus participating in the initiation and progression of periodontitis [38, 39]. The association between *D. pneumosinte* and AD progression requires further investigation.

### Features of the GCF metabolome

In periodontal tissue, GCF is a type of noninvasive peripheral body fluid from blood serum that is anatomically closer to the cerebrospinal fluid and blood–brain barrier. Therefore, diagnostic and prognostic biomarker analyses using GCF have the potential to detect pathological changes in patients with AD. In our study, we first screened 165 differentially abundant metabolites in GCF samples using LC–MS/MS among the three groups. After multiomics DIABLO, we further focused on 19 differentially abundant metabolites (Table S3), which were significantly correlated with five different species (*V. parvula*, *D. pneumosintes*, *L. buccalis*, *P. goodfellowii*, and *A. massiliensis*), and these metabolites possibly separated AD patients from cognitively normal people. Changes in some differentially abundant metabolites, including galactinol from galactose metabolism and adenine and deoxyadenosine from the purine metabolism pathway, also implicated an association with AD progression.

First, as a key source of energy and a crucial structural element in complex molecules, galactose metabolism is particularly important for organisms [40]. Galactose metabolism disorders may affect brain development and even cognitive function. Recent studies have also shown that d-galactose can cause memory and learning deficits in rats as a senescence agent and can be used to establish cognitive impairment models in vivo [41]. However, the role of galactinol in cognitive function has not been reported. Second, the synthesis, degradation, and interconversion of DNA, RNA, lipids, proteins, and carbohydrates all require purine metabolism. Purine and purine nucleosides are important sources of nucleic acid biosynthesis in bacteria and hosts, and adenine nucleoside triphosphate synthesized from adenine and adenosine is the most direct energy source in organisms. Recent studies have shown that AD progression is closely related to purine metabolism and the downstream signalling of purinergic receptors [42]. Purinergic receptors are increased in degenerating neurons and around β-amyloid plaques and play an important role in regulating the level of neuroinflammation [43]. It has also been shown that the expression of purinergic receptors in endothelial cells of the blood–brain barrier and microglia increases in AD patients, which may reflect neuroinflammation caused by infection and activation of microglia in the AD brain [44]. Metabolites from the periphery, such as periodontal tissue, may also enter the brain. Therefore, purinergic receptors have become a new target for the treatment of AD in recent years [43]. Finally, it has been shown that anxiety, depression, and memory impairment induced by d-galactose could be protected by adenosine through its antioxidant and neuromodulatory effects in rats, indicating that cognitive function may be affected by multiple metabolic pathways, which needs more exploration.

### Oral-brain axis: host-microbial interactions on linking the periodontitis to AD progression

Recently, the concept of the oral-brain axis has gained substantial traction [3, 7]. Accumulating evidence suggests that microbial dysbiosis or infections may directly influence AD pathogenesis through the nervous system and blood circulation pathways or indirectly through immune inflammation, endocrine, and metabolic pathways [45–48]. As the second largest microbiota in the human body, the oral microbiota may affect the pathological process of AD by activating microglia to induce inflammation or release metabolites [11]. As an important part of the oral microbiota, the subgingival microbiota and their metabolic products may affect the pathological process of AD through microbial dysbiosis and metabolic disorders (Figure S6), which may also have the potential to prevent and diagnose AD.

From the results, there were distinct differences in clinical data, the microbial community of subgingival plaque, and metabolites of GCF among the cognitively normal group and the aMCI and AD groups. Specifically, most cognitive impairment-upregulated metabolites showed a significantly positive relationship with some enriched species in those with cognitive impairment and a significantly negative relationship with enriched species in cognitively normal subjects. Similarly, downregulated metabolites in those with cognitive impairment had an opposite relationship with species. These results confirm that host and periodontal microorganisms are closely related and interact with AD progression. This result suggests that the microbiota and metabolites might be useful tools for predicting AD progression in the future. In the current study, an ROC curve was used to evaluate how these differentially abundant metabolites indicated or further predicted AD progression [49, 50]. The results showed that five differentially abundant metabolites, namely, galactinol, sn-glycerol 3-phosphoethanolamine, D-mannitol, 1 h-indole-1-pentanoic acid, 3-(1-naphthalenylcarbonyl)-, and L-iditol, distinguished the AD group from the CN and aMCI groups and yielded satisfactory accuracy, but these indicators require further investigation to demonstrate their clinical significance.

### Limitations

However, our study still has certain limitations. First, the prevalence of periodontal health is 5.0% in the 55–64-year-old group and 9.3% in 65–74-year-old group in China, and the prevalence of periodontitis is 69.3 and 64.6%, respectively. Moreover, the severity of periodontal disease is positively correlated with age [51]. Therefore, age-matched periodontally healthy and cognitively normal control subjects were not successfully recruited for this study. Second, statistical studies have shown that the prevalence and mortality of AD in the Chinese Han population increases with age, and the prevalence in females is higher than that in males [52–54]. Due to the significantly higher mortality in elderly female patients with AD than in males and the age-related prevalence [55], the mean age of the AD and aMCI groups was higher than that of cognitively normal controls in this smaller sample size study, and there were significantly more male patients than female patients in the AD group. Therefore, the AUC values and diagnostic abilities of potential biomarkers may be affected by age and sex. Third, it was only a hospital-based cross-sectional study rather than a longitudinal cohort, and we could not obtain information regarding the correlations with the species/metabolites and conversion of clinical phenotypes (from CN to aMCI/AD and aMCI to AD). Further investigations, especially follow-up cohorts consisting of larger sample sizes and age- and sex-matched participants, are warranted to validate our results.

## Conclusion

This is the first combined subgingival microbiome and GCF metabolome study in patients with AD and aMCI, which revealed that periodontal microbial dysbiosis and metabolic disorders may be involved in the etiology and progression of AD, and the differential abundance of the periodontal microbiota and metabolites correlated with cognitive function may be useful as potential markers for AD in the future.

### Supplementary Information

**Additional file 1: Figure S1.** Heat plot of the differential metabolites and pathways among CN, aMCI and AD groups. **Figure S2.** Histogram of metabolite set enrichment analysis and topology analysis of differentially abundant metabolites. **Figure S3.** Correlations between subgingival plaque bacteria (species level) and metabolites in GCF in CN and aMCI group. **Figure S4.** Correlations between subgingival plaque bacteria (species level) and metabolites in GCF in aMCI and AD group. **Figure S5.** Correlations between subgingival plaque bacteria (species level) and metabolites in GCF in CN and AD group. **Figure S6.** Oral-brain axis. **Table S1.** Subgingival microbiome community significantly correlated with cognitive function. **Table S2.** Differential metabolic pathways and included differential metabolites among the AD, aMCI and CN groups. **Table S3.** Candidate diagnostic metabolites among the AD, aMCI and CN groups. **Table S4.** The AUC of the candidate diagnostic biomarkers. 

## Data Availability

The raw data supporting the conclusion of this manuscript will be made available by the authors, without undue reservation, to any qualified researcher.

## Abbreviations

AD: Alzheimer’s disease
aMCI: Amnestic mild cognitive impairment
GCF: Gingival crevicular fluid
CN: Cognitively normal
rRNA: Ribosomal RNA
LC–MS/MS: Liquid chromatography linked to tandem mass spectrometry
DIABLO: Data integration analysis for biomarker discovery using latent components
MoCA: Montreal Cognitive Assessment
MMSE: Mini-Mental State Examination
*ApoE*: Apolipoprotein E
AV45 PET: 8F-florbetapir (AV45) positron emission tomography
PPD: Periodontal probing depth
CAL: Clinical attachment loss
PLI: Plaque index
BOP: Bleeding on probing
GI: Gingival index
PCR: Polymerase chain reaction
ASV: Amplicon sequence variant
PCoA: Principal coordinate analysis
LEfSe: Linear discriminant analysis effect size
MaAsLin2: Microbiome multivariable associations with linear models
PCA: Pareto-scaled principal component analysis
PLS-DA: Partial least-squares discriminant analysis
KEGG: Kyoto Encyclopedia of Genes and Genomes
AUC: Area under the curve
ROC: Receiver operating characteristic
SD: Standard deviation
ANOVA: Analysis of variance
