# Supplementary material for: Profiles of subgingival microbiomes and gingival crevicular metabolic signatures in patients with amnestic mild cognitive impairment and Alzheimer’s disease
Source: Alzheimers Res Ther. 2024 Feb 19;16:41. doi: 10.1186/s13195-024-01402-1 (PMC10875772; doi:10.1186/s13195-024-01402-1)
Supplement: Supplementary file 1 — Additional file 1: Figure S1. Heat plot of the differential metabolites and pathways among CN, aMCI and AD groups. Figure S2. Histogram of metabolite set enrichment analysis and topology analysis of differentially abundant metabolites. Figure S3. Correlations between subgingival plaque bacteria (species level) and metabolites in GCF in CN and aMCI group. Figure S4. Correlations between subgingival plaque bacteria (species level) and metabolites in GCF in aMCI and AD group. Figure S5. Correlations between subgingival plaque bacteria (species level) and metabolites in GCF in CN and AD group. Figure S6. Oral-brain axis. Table S1. Subgingival microbiome community significantly correlated with cognitive function. Table S2. Differential metabolic pathways and included differential metabolites among the AD, aMCI and CN groups. Table S3. Candidate diagnostic metabolites among the AD, aMCI and CN groups. Table S4. The AUC of the candidate diagnostic biomarkers. [file 13195_2024_1402_MOESM1_ESM.docx]

**Supplementary Material**

**
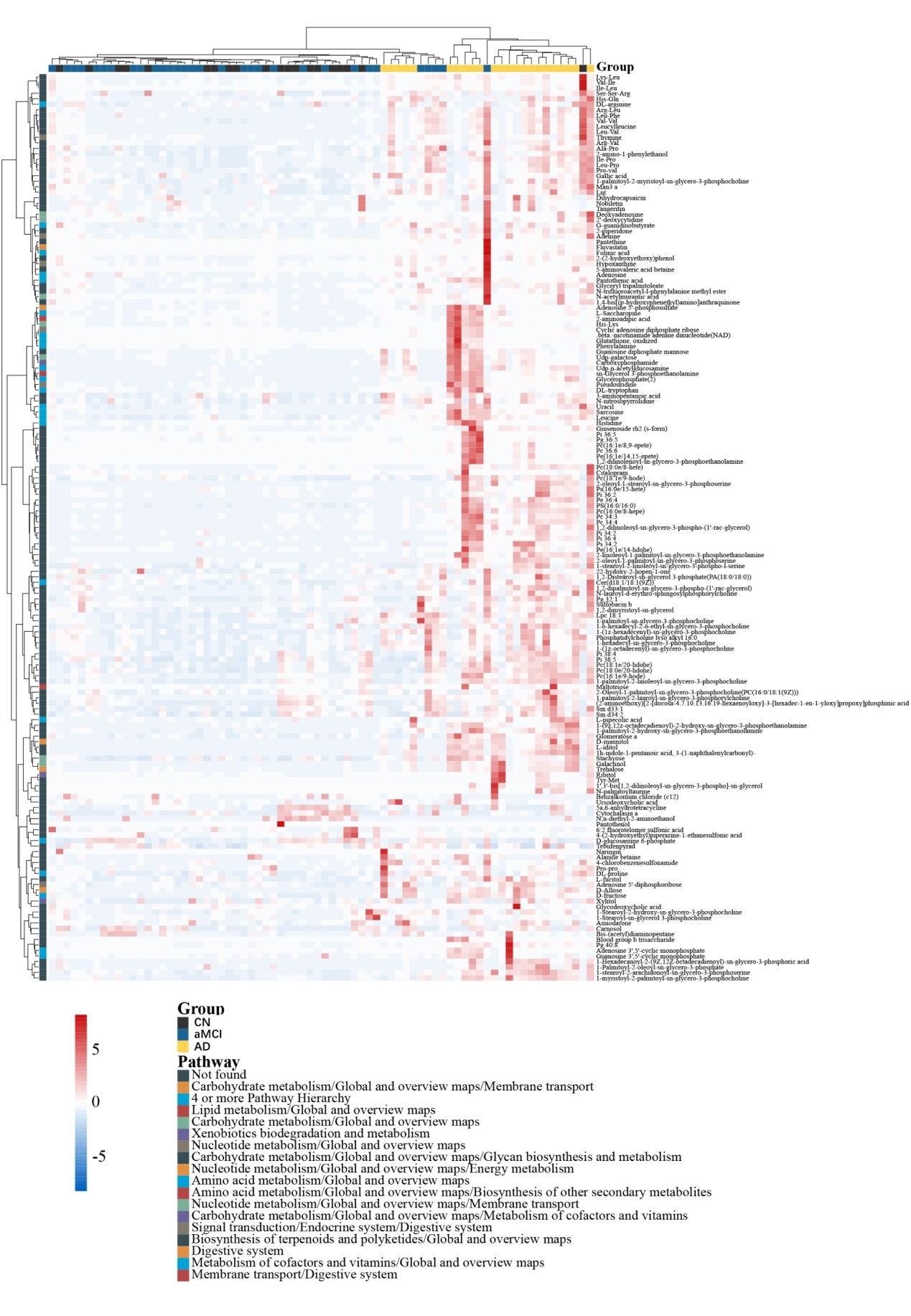
**

**Figure S1 Heat plot of the differential metabolites and pathways among CN, aMCI and AD groups.** The differential metabolites were determined using MaAsLin2. Only the metabolites with FDR-corrected *P* value <0.25 were shown.

**Figure S2** **Histogram of metabolite set enrichment analysis and topology analysis of differentially abundant metabolites.** The X-axis indicates the ratio of differentially abundant metabolites to the total number of metabolites that were included in the pathway (KEGG database-matched results), and the bar colour indicates the *P* value in the enrichment analysis.


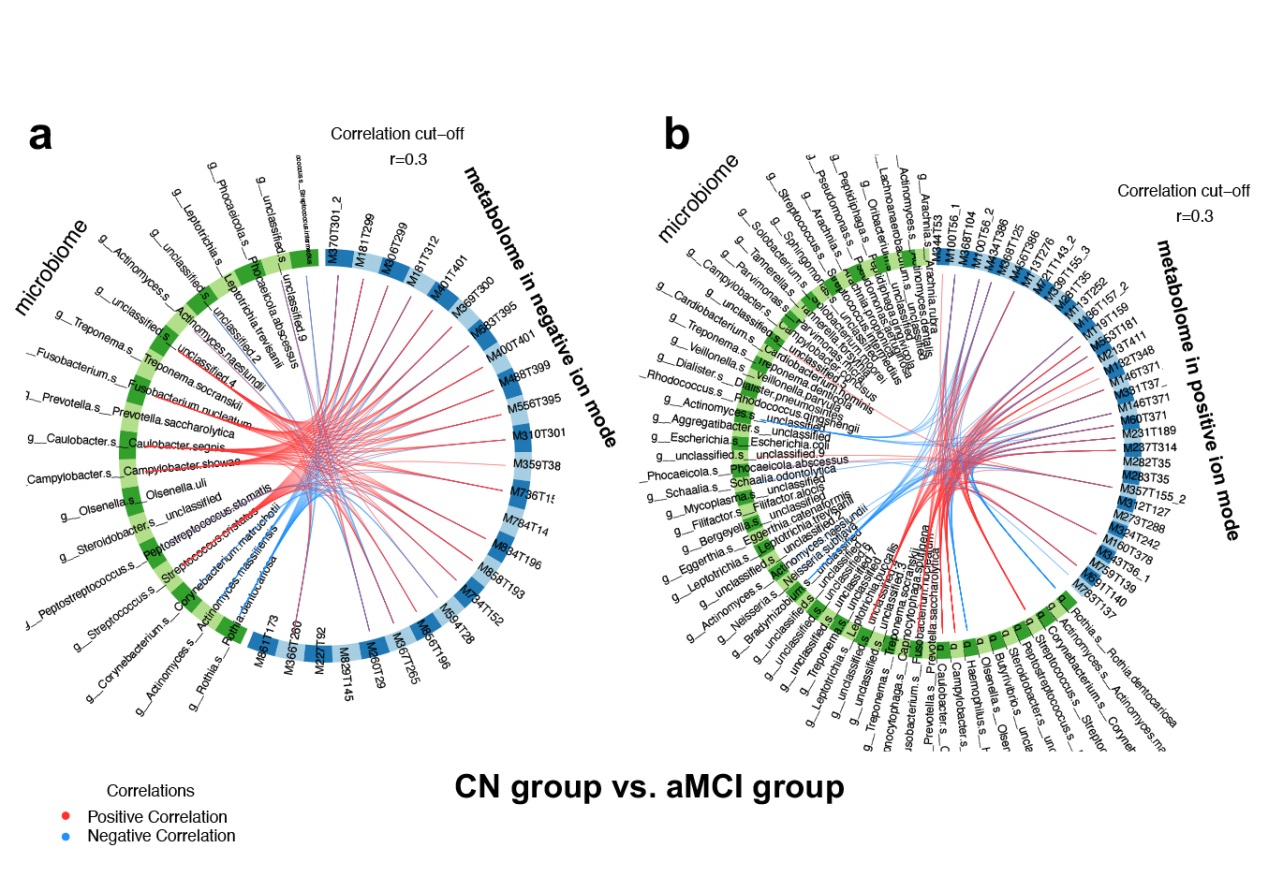


**Figure S3 Correlations between subgingival plaque bacteria (species level) and metabolites in GCF in CN and aMCI group.** (**a**) Correlation model between subgingival microbiome and metabolome in negative ion mode. (**b**) Correlation model between subgingival microbiome and metabolome in positive ion mode.


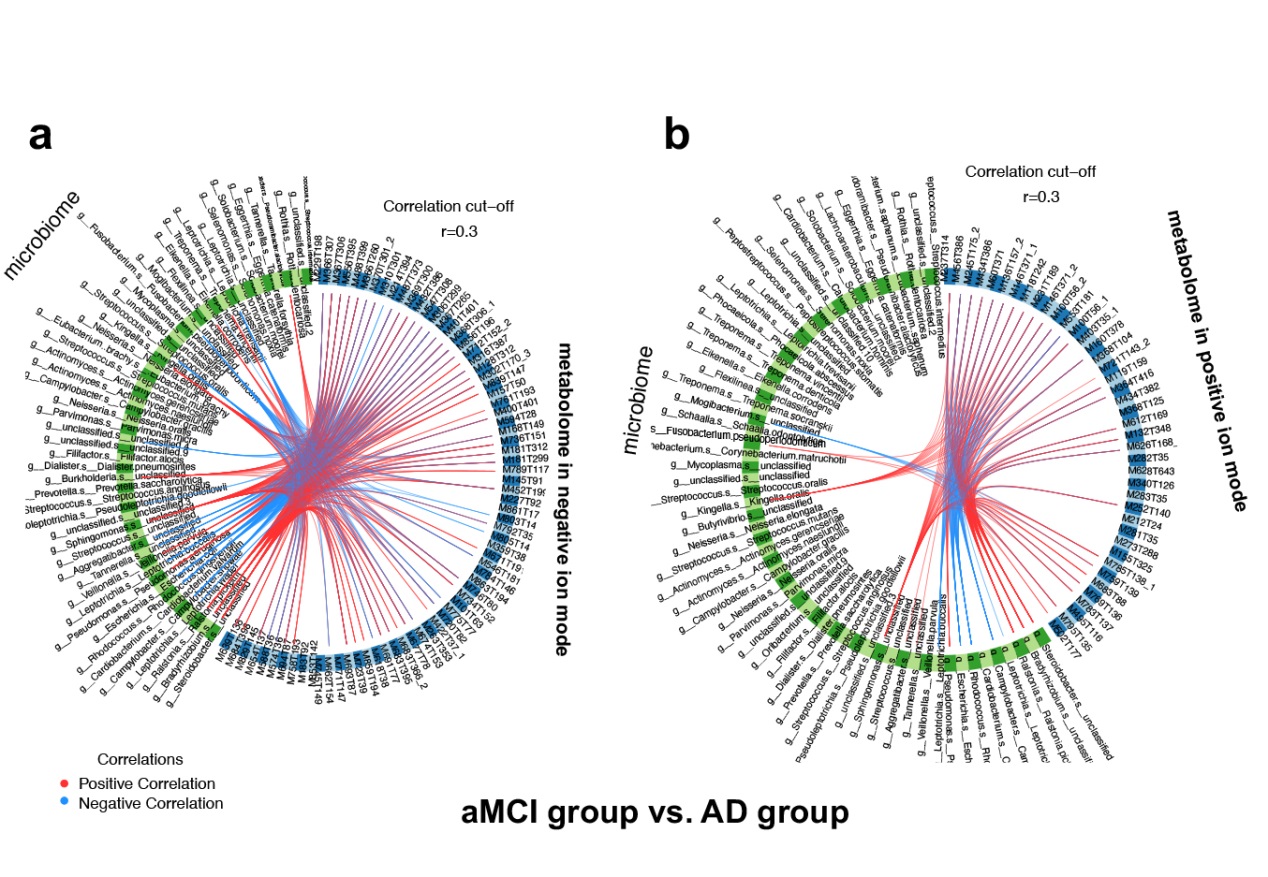


**Figure S4 Correlations between subgingival plaque bacteria (species level) and metabolites in GCF in aMCI and AD group.** (**a**) Correlation model between subgingival microbiome and metabolome in negative ion mode. (**b**) Correlation model between subgingival microbiome and metabolome in positive ion mode.


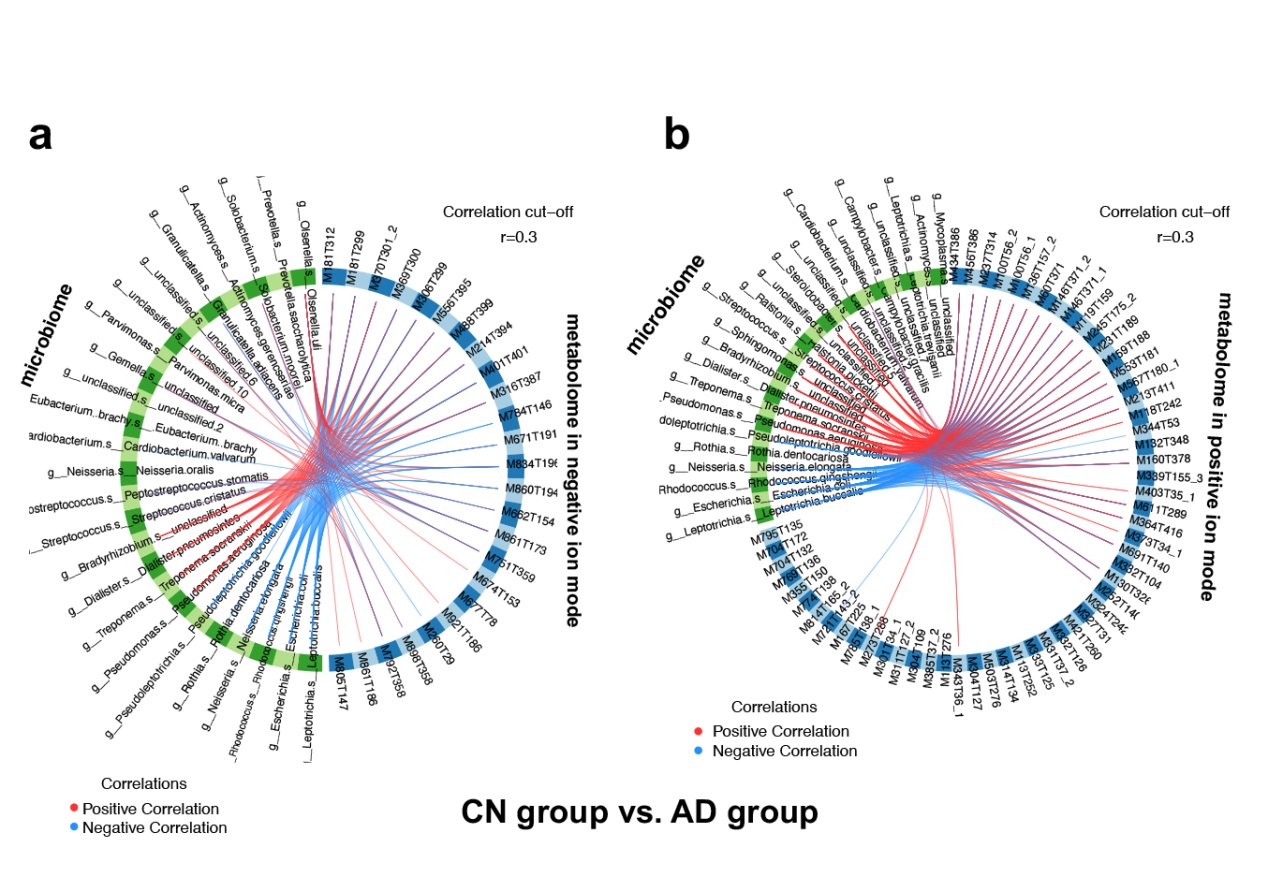


**Figure S5 Correlations between subgingival plaque bacteria (species level) and metabolites in GCF in CN and AD group.** (**a**) Correlation model between subgingival microbiome and metabolome in negative ion mode. (**b**) Correlation model between subgingival microbiome and metabolome in positive ion mode.


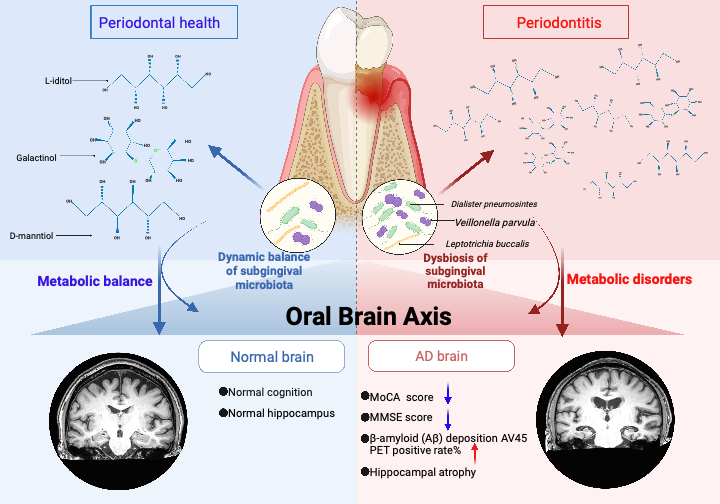


**Figure S6 Oral-brain axis.** Periodontal subgingival microbial dysbiosis and metabolic disorder may affect the pathological process of Alzheimer’s disease.

**Table S1 Subgingival microbiome community significantly correlated with cognitive function.**

| **Taxon (species level)** | **Coefficient** | **FDR-corrected *P* value** |  |
| --- | --- | --- | --- |
| *Lancefieldella parvula* | -0.00156784 | 0.028 | Negative correlation with cognitive function |
| *Prevotella melaninogenica* | -0.00882783 | 0.074 |  |
| *Megasphaera micronuciformis* | -0.00333092 | 0.084 |  |
| *Anaeroglobus geminatus* | -0.00512537 | 0.086 |  |
| *Veillonella parvula* | -0.0364545 | 0.100 |  |
| *Streptococcus anginosus* | -0.00626201 | 0.147 |  |
| *Campylobacter gracilis* | -0.00810334 | 0.148 |  |
| *Dialister pneumosintes* | -0.00190308 | 0.230 |  |
| *[Eubacterium] yurii* | 0.00178552 | 0.072 | Positive correlation with cognitive function |
| *Pseudoleptotrichia goodfellowii* | 0.000676977 | 0.075 |  |
| *Campylobacter rectus* | 0.00151813 | 0.086 |  |
| *Leptotrichia buccalis* | 0.00943149 | 0.100 |  |
| *Streptococcus sanguinis* | 0.00687583 | 0.147 |  |
| *Actinomyces massiliensis* | 0.00185133 | 0.148 |  |
| *Haemophilus parainfluenzae* | 0.0111592 | 0.220 |  |
| *Campylobacter concisus* | 0.00369048 | 0.220 |  |

Note: The correlations between microbiome and disease severity were determined using MaAsLin2. Only the species with FDR-corrected *P* value <0.25 were shown

**Table S2 Differential metabolic pathways and included differential metabolites among the AD, aMCI and CN groups.**

| **ID** | **Pathway (Size*)** | ***P*-value** | **Differential metabolites** |
| --- | --- | --- | --- |
| map00230 | Purine metabolism (7) | 6.24E-02 | Adenine, Hypoxanthine, Adenosine, Deoxyadenosine, Adenosine 5'-phosphosulfate, Adenosine 5'-diphosphoribose, Guanosine 3',5'-cyclic monophosphate |
| map00520 | Amino sugar and nucleotide sugar metabolism (4) | 1.43E-01 | D-glucosamine 6-phosphate, D-fructose, Uridine diphosphategalactose, Guanosine diphosphate mannose |
| map00310 | Lysine degradation (3) | 1.56E-01 | L-Saccharopine, 2-aminoadipic acid, L-pipecolic acid |
| map00052 | Galactose metabolism (4) | 1.84E-01 | Uridine diphosphategalactose, D-fructose, Stachyose, Galactinol |
| map00400 | Phenylalanine, tyrosine and tryptophan biosynthesis (1) | 2.03E-01 | L-Phenylalanine |
| map00240 | Pyrimidine metabolism (3) | 3.66E-01 | 2'-deoxycytidine, Uracil, Thymine |
| map00970 | Aminoacyl-tRNA biosynthesis (3) | 5.01E-01 | L-Histidine, L-Phenylalanine, L-Leucine |

*Size, the number of differential metabolites was included in the pathway(KEGG database matched result)

**Table S3 Candidate diagnostic metabolites among the AD, aMCI and CN groups**

| **ID** | **Name** | **Adducts** | **FC** | **FDR-corrected p-value** |
| --- | --- | --- | --- | --- |
| M252T140 | Deoxyadenosine | [M+H]+ | 2.14 (AD vs. CN) | 0.233 (AD vs. CN) |
| M130T326 | DL-arginine | [M+H-CH3ON]+ | 1.54 (AD vs. CN) | 0.087 (AD vs. CN) |
| M132T348 | Alanine betaine | [M+H]+ | 7.28 (AD vs. CN) | 0.018 (AD vs. CN) |
| M118T242 | N,n-diethyl-2-aminoethanol | [M+H]+ | 0.16 (AD vs. CN) | 0.011 (AD vs. CN) |
| M213T411 | Pro-pro | [M+H]+ | 1.94 (AD vs. aMCI)  3.77 (AD vs. CN) | 0.057 (AD vs. aMCI)  0.005 (AD vs. CN) |
| M160T378 | 5-aminovaleric acid betaine | [M+H]+ | 0.91 (AD vs. aMCI)  4.73 (AD vs. CN) | 0.242 (AD vs. aMCI)  0.025 (AD vs. CN) |
| M119T159 | 2-(2-hydroxyethoxy)phenol | [M+H-2H2O]+ | 1.43 (AD vs. aMCI)  3.50 (AD vs. CN) | 0.026 (AD vs. aMCI)  0.002 (AD vs. CN) |
| M245T175_2 | Leucyl leucine | [M+H]+ | 1.91 (AD vs. aMCI)  2.02 (AD vs. CN) | 0.045 (AD vs. aMCI)  0.002 (AD vs. CN) |
| M231T189 | Leu-Val | [M+H]+ | 1.76 (AD vs. aMCI)  2.07 (AD vs. CN) | 0.046 (AD vs. aMCI)  0.002 (AD vs. CN) |
| M136T157_2 | Adenine | [M+H]+ | 1.82(AD vs. aMCI)  4.02 (AD vs. CN) | 0.034 (AD vs. aMCI)  0.000 (AD vs. CN) |
| M100T56_2 | 2-piperidone | [M+H]+ | 1.90(AD vs. aMCI)  4.40 (AD vs. CN) | 0.011 (AD vs. aMCI)  0.000 (AD vs. CN) |
| M146T371_2 | G-guanidinobutyrate | [M+H]+ | 1.87(AD vs. aMCI)  2.94 (AD vs. CN) | 0.040 (AD vs. aMCI)  0.000 (AD vs. CN) |
| M434T386 | Fluvastatin | [M+Na]+ | 0.86(AD vs. aMCI)  6.72 (AD vs. CN) | 0.034 (AD vs. aMCI)  0.000 (AD vs. CN) |
| M456T386 | Folinic acid | [M+H-H2O]+ | 1.13(AD vs. aMCI)  5.20 (AD vs. CN) | 0.034(AD vs. aMCI)  0.000 (AD vs. CN) |
| M401T401 | Galactinol | (M+CH3COO)- | 17.76(AD vs. aMCI)  28.68 (AD vs. CN) | 0.000 (AD vs. aMCI)  0.000 (AD vs. CN) |
| M214T394 | sn-Glycerol 3-phosphoethanolamine | [M-H]- | 19.64 (AD vs. aMCI)  30.01 (AD vs. CN) | 0.000 (AD vs. aMCI)  0.000 (AD vs. CN) |
| M181T312 | D-mannitol | [M-H]- | 121.39 (AD vs. aMCI)  185.05 (AD vs. CN) | 0.000 (AD vs. aMCI)  0.000 (AD vs. CN) |
| M370T301_2 | 1 h-indole-1-pentanoic acid, 3-(1-naphthalenylcarbonyl)- | [M-H]- | 54.70 (AD vs. aMCI)  78.78 (AD vs. CN) | 0.000 (AD vs. aMCI)  0.000 (AD vs. CN) |
| M181T299 | L-iditol | [M-H]- | 135.79 (AD vs. aMCI)  150.75 (AD vs. CN) | 0.000 (AD vs. aMCI)  0.000 (AD vs. CN) |

Abbreviations: FC, fold change.

Note: Candidate diagnostic biomarkers were determined using MaAsLin2 (FDR-corrected *P* value <0.25) and discriminant analysis with DIABLO (Correlation coefficient r>0.3)

**Table S4 The AUC of the candidate diagnostic biomarkers**

| **Name** | **AD vs. aMCI** | **AD vs. CN** | **aMCI vs. CN** | **AD & aMCI vs. CN** |
| --- | --- | --- | --- | --- |
| **Metabolites** | | | | |
| Deoxyadenosine | 0.6655 | 0.7424 | 0.5532 | 0.6338 |
| DL-arginine | 0.6381 | 0.6772 | 0.5290 | 0.5921 |
| Alanine betaine | 0.6508 | 0.7413 | 0.5782 | 0.6476 |
| N,n-diethyl-2-aminoethanol | 0.5694 | 0.7804 | 0.7460 | 0.7606 |
| Pro-pro | 0.7027 | 0.8435 | 0.6589 | 0.7375 |
| 5-aminovaleric acid betaine | 0.6670 | 0.7630 | 0.5605 | 0.6408 |
| 2-(2-hydroxyethoxy)phenol | 0.7468 | 0.8522 | 0.5766 | 0.6940 |
| Leucyl leucine | 0.7251 | 0.8804 | 0.6226 | 0.7324 |
| Leu-Val | 0.7272 | 0.8891 | 0.6210 | 0.7352 |
| Adenine | 0.7482 | 0.8750 | 0.6234 | 0.7306 |
| 2-piperidone | 0.8156 | 0.8663 | 0.6008 | 0.7139 |
| G-guanidinobutyrate | 0.7489 | 0.8391 | 0.5952 | 0.6991 |
| Fluvastatin | 0.7924 | 0.9304 | 0.6476 | 0.7681 |
| Folinic acid | 0.7945 | 0.9304 | 0.6621 | 0.7764 |
| Galactinol | 0.9762 | 0.9815 | 0.6266 | 0.7778 |
| sn-Glycerol 3-phosphoethanolamine | 0.9832 | 0.9902 | 0.5855 | 0.7579 |
| D-mannitol | 0.9846 | 0.9924 | 0.5589 | 0.7435 |
| 1 h-indole-1-pentanoic acid, 3-(1-naphthalenylcarbonyl)- | 0.9881 | 0.9848 | 0.5855 | 0.7556 |
| L-iditol | 0.9867 | 0.9859 | 0.5508 | 0.6778 |
| **Microbes** | | | | |
| *Actinomyces. massiliensis* | 0.5259 | 0.5109 | 0.5484 | 0.5231 |
| *Leptotrichia. buccalis* | 0.5947 | 0.5913 | 0.5161 | 0.5481 |
| *Pseudoleptotrichia. goodfellowii* | 0.5750 | 0.5348 | 0.5339 | 0.5046 |
| *Dialister. pneumosintes* | 0.6143 | 0.7370 | 0.6048 | 0.6611 |
| *Veillonella. parvula* | 0.5175 | 0.6870 | 0.6403 | 0.6602 |
